# Effect of faculty and near-peer mentoring for managing burnout among Indian medical student

**DOI:** 10.6026/973206300220542

**Published:** 2026-01-31

**Authors:** Puja Singh, Sarika Chouhan, Sonal Kulshreshtha

**Affiliations:** 1 Department of Pathology, Bundelkhand Medical College, Sagar, Madhya Pradesh, India; 2 2Department of Ophthamology, Bundelkhand Medical College, Sagar, Madhya Pradesh, India; 3 Department of Obstetrics & Gynaecology, Gajra Raja Medical College, Gwalior, Madhya Pradesh, India

**Keywords:** Burnout, faculty mentorship, near-peer mentorship, mentor, educational education

## Abstract

Burnout among medical students leads to emotional exhaustion and diminished academic performance. Therefore, it is of interest to explore comparative performance of near-peer and faculty mentorship in alleviating the burnout. Participants were divided into three groups (no mentorship, near-peer, and faculty mentorship) and assessed for burnout through the Maslach Burnout Inventory before and after a 6-month intervention. Data shows that female students had higher baseline burnout levels than males, and hostel residents experienced greater burnout than those living with family. We found that both mentorship types significantly alleviated burnout. Near- peer mentorship notably decreased emotional exhaustion and isolation due to enhanced relatability, while faculty mentorship improved academic efficacy and emotional exhaustion. Thus, both mentorship approaches play vital, complementary roles in reducing burnout among medical students.

## Background:

Medical students frequently encounter challenging situations during their academic journey, which can contribute to mental health issues. Burnout affects not only their academic performance but also their overall well-being and professional development [1]. This emotional experience is often triggered when the demanding path to becoming a physician outweighs its inherent pitfalls and stressors. The rigorous curricula, demanding examinations, and other external stressors characteristic of medical education are recognized as major contributors to burnout [2]. The prevalence of burnout among medical students is heterogeneous, ranging from 37.23% to as high as 88%, and varying by the year of study [3, 4]. Burnout can manifest as emotional exhaustion, depersonalization, and reduced personal accomplishment, leading to negative consequences such as worsened academic engagement, feelings of inadequacy, and an elevated risk of depression and suicide [5]. Mentorship has emerged as a crucial strategy in addressing student burnout [6]. Traditionally, mentorship to medical students is offered by faculty members. This arrangement is prone to a generation gap and unease, inhibitive interaction, and a lack of a realistic comprehension of the struggles of students [7]. Near-peer mentoring, offered by senior students, can be particularly effective. Such programs can effectively enhance resilience and support mental health, thereby contributing to the development of skilled healthcare professionals [8, 9]. Therefore, it is of interest to evaluate the impact of near-peer mentoring on burnout among medical students by comparing it with faculty-led mentoring and no mentoring.

## Methodology:

This comparative, quasi-experimental, and prospective study was conducted at Bundelkhand Government Medical College, Sagar, India, for duration of 6 months from Sep 2024. Due ethical approval taken from institute ethical committee and written informed consent was obtained from all participants. Mentee Selection: One hundred twenty MBBS students volunteered for the study. Students were informed that their decision to participate would not affect their academic standing. Students who did not tender their consent or with pre- existing psychiatric diagnoses or those receiving counselling for stress were exclude from the study.

## These students were divided into the following three groups:

[1] Groups A (Control): 40 students: Did not receive any mentorship during the study period

[2] Groups B: 40 students: Assigned faculty mentorship

[3] Groups C: 40 students: Assigned near-peer mentorship

## Mentor selection:

The following two groups of mentors were selected:

1) Faculty Mentors: A group of 10 volunteer faculty members was selected.

2) Near-peer Mentors: A group of 20 volunteer second-year MBBS students who demonstrated strong academic standing and interpersonal skills.

All mentors underwent a standardized one-day workshop focused on active listening, setting boundaries, recognizing signs of distress, and providing resources without engaging in psychotherapy. To minimise any biases, all the allocations within the groups were kept random. Intervention: The control group (group A) continued routine academic activities without structured mentoring.

## The intervention groups (group B & C) then participated in the corresponding mentoring program, scheduled as below:

[1] Initial Pairing: Each faculty mentor was paired with 4 mentees & near-peer mentor was paired with 2 mentees.

[2] Structured Contact: Mandatory bi-monthly small group meetings for the first three months, transitioning to monthly meetings for the remainder of the academic year.

[3] Unstructured Support: Mentors were available for ad-hoc support.

[4] Thematic Focus: Meetings covered pre-defined topics such as study strategies, time management, work-life integration, and navigating clinical exposures, based on a curriculum developed by faculty and student wellness advisors.

## Data collection:

At baseline (week 0), all groups completed the MBI-GS(S) and demographic questionnaire. At the end of six months (week 24), all groups again completed the MBI-GS(S).

Feedbacks were collected from participants, faculty, and near-peer mentors.

[1] Sociodemographic Proforma: Included details such as age, gender, academic year, residence (hostel/family), and participation in extracurricular activities.

[2] Mind Garden's Maslach Burnout Inventory-General Student Survey (MBI-GS(S)): This instrument measures three components of burnout: Emotional Exhaustion (EE), Cynicism (CY), and Academic Efficacy (AE). It contains 16 items rated on a 7-point Likert scale (0 = never to 6 = always). Higher EE and CY scores, along with lower AE scores, indicate higher levels of burnout.

[3] Mentorship Evaluation Questionnaire: Administered, using Google Forms, at the end of the program to assess satisfaction, perceived benefits, and qualitative feedback regarding mentor-mentee interaction.

## Statistical analysis:

Statistical analysis was performed using Python (v3.9), Pandas (v1.3.5) and Seaborn (v0.11.2). Sentiment analysis was performed, using a Word cloud on the feedback received from participants. A p-value < 0.05 was considered statistically significant.

## Results/observations:

**Figure 1:**
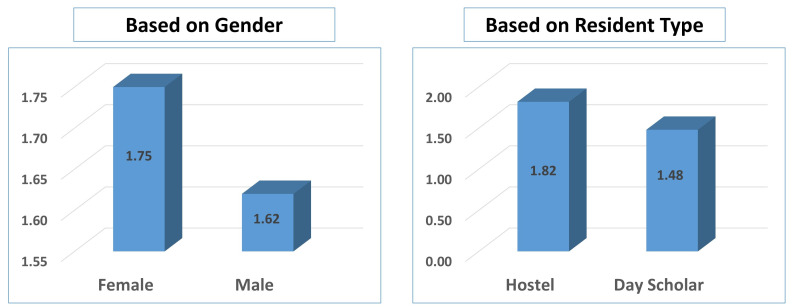
Impact of demographic factors, using overall scores obtained by MBI-GS(S), Gender, and Resident Type, on Burnout of participants (n=120)

**Figure 2:**
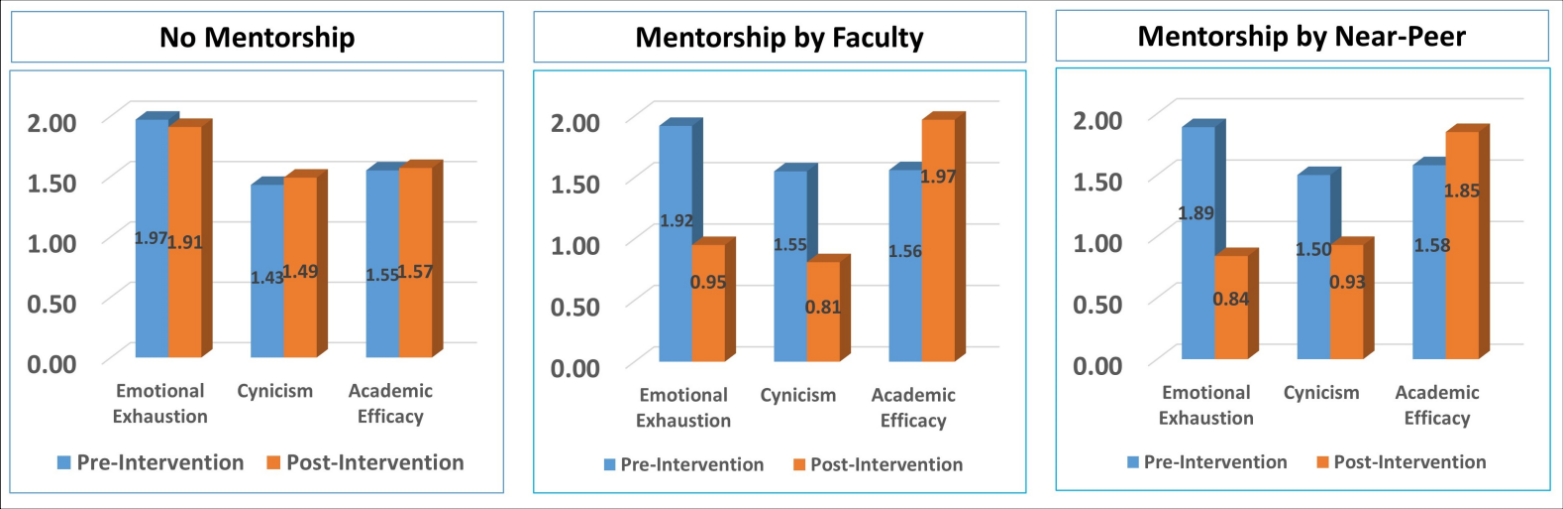
Comparison of scores obtained using MBI-GS(S) across different study groups (n=40)

**Figure 3:**
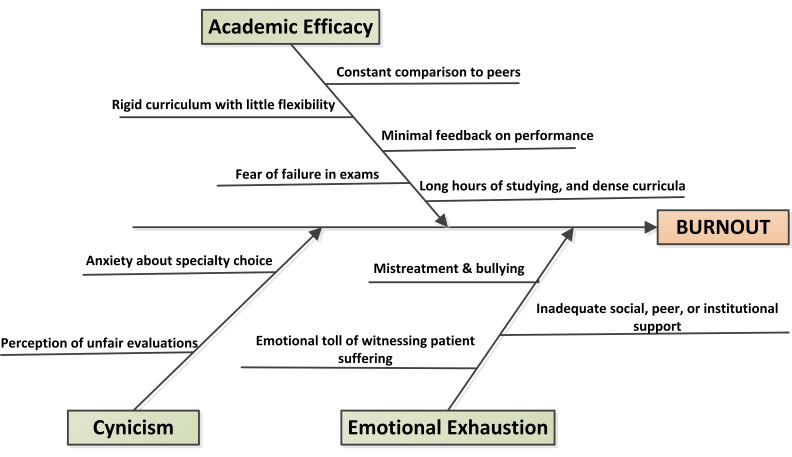
Fish-bone diagram of burnout causes among students

**Figure 4:**
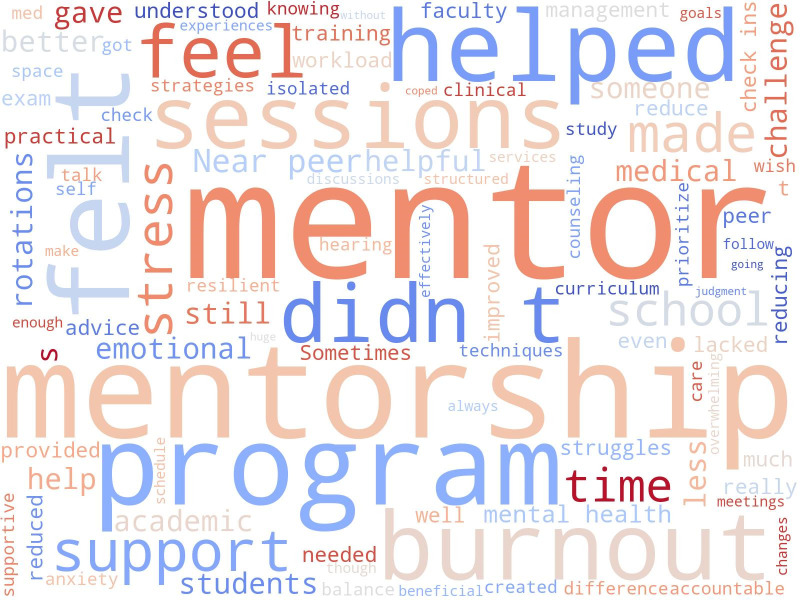
Sentiment analysis using word cloud based on student's feedback

Out of 120 MBBS students who participated in the study, 78 (65%) were male. Of these participants, 107 (89.17%) stayed in hostels, while the remaining 13 were day scholars. The impact of demographic factors on the participants' burnout is presented in Figure 1. Females have shown significantly higher burnout, 1.75, as compared to males, 1.62. Similarly, those who were staying in the hostel demonstrated higher burnout, 1. 82, as compared to those staying with the guardians, 1.48. For Group A, a negligible change was observed for EE, CY, and AE. For groups B and C, faculty and near-peer mentorship led to a sharp drop in EE and CY, and a significant rise for AE. Overall, faculty mentorship was more effective as compared to near-peer mentorship; however, near-peer mentorship performed better for controlling emotional exhaustion. Comparative analysis of the interventions on three groups is presented in Figure 2. As illustrated in Figure 3, the fishbone diagram, the analysis of factors contributing to burnout among medical students reveals that burnout primarily stems from three interrelated domains: academic inefficacy, cynicism, and emotional exhaustion. The word cloud generated from student feedback, Figure 4, revealed that faculty and near-peer mentorship programs significantly enhanced emotional well-being, reduced stress and burnout, and fostered a sense of support and understanding among medical students. Participants appreciated guidance on coping strategies, mental health, and academic challenges. The mentorship sessions were perceived as beneficial in improving resilience and confidence. However, some students reported limitations such as time constraints, irregular meetings, and insufficient follow-up, indicating the need for more structured, consistent, and frequent mentorship interactions to maximize program effectiveness.

## Discussion:

Medical school is a demanding academic environment that exposes students to significant mental and physical challenges, which can lead to burnout. This issue is particularly relevant for medical students who face curricular challenges, relocation, and separation from family. Some studies suggest that the risk of burnout can emerge early in medical training, even within the first year of study. In this study, demographic factors, in line with other studies, showed notable effects: female medical students reported higher levels of emotional exhaustion, depersonalization, and perceived stress than males [1, 4]. Meanwhile, students living in hostels experienced more burnout than those living with family, likely due to fewer emotional supports and greater environmental stressors - consistent with recent findings [10, 11]. The absence of mentorship failed to bring about significant psychological or academic benefit. Participants largely retained pre-existing levels of burnout and detachment. As per this study, faculty mentorship appears to be the most effective intervention in enhancing psychological well-being and academic engagement. The authoritative structured guidance, professional insights, and validation from experienced mentors likely contributed to these improvements. Several contemporary reviews emphasize that faculty mentorship contributes to mentees' academic efficacy and career development while also requiring institutional recognition and structured training to be effective [12, 13]. Comparatively, near-peer mentoring produced more immediate reductions in emotional exhaustion and feelings of isolation, whereas faculty mentorship tended to influence longer-term professional outcomes and helped reduce uncertainty about specialty choice and performance expectations [12]. These complementary roles suggest a scaffolder model (near-peer for immediate psychosocial buffering; faculty for career and longitudinal professional support) is likely to yield the greatest impact on early-phase burnout [14, 15]. This complementarity is supported by studies recommending tiered or mosaic mentoring frameworks combining peers and senior faculty. Limitations observed in both models were primarily logistical: inconsistent meeting schedules, limited contact time, and variable mentor availability decreased perceived effectiveness. Some mentees reported that near-peer mentors, while empathetic, lacked the authority or experience to resolve systemic issues; faculty mentors, while experienced, were sometimes perceived as less approachable or too time-limited to provide regular psychosocial support. These trade-offs underline the need for clearly defined roles, expectations, and hybrid pathways enabling escalation from peer to faculty support when required [16]. From the fish-bone diagram and word cloud, it's very clear that burnout among medical students is multifactorial, arising from the combined effects of academic overload, emotional stressors, and systemic issues within the educational environment. Addressing these root causes through curriculum reform, mentorship programs, fair evaluation systems, and enhanced psychological support can help mitigate burnout and improve student well-being.

## Limitations:

The study was a single institution with small sample, which may restrict the generalizability of findings. Burnout and well-being outcomes were based on self-reported questionnaires, which are prone to biases. The study primarily assessed immediate or short-term outcomes and long-term effects on resilience, academic performance, and sustained burnout reduction were not examined. The effectiveness of mentoring sessions may have varied depending on mentors' interpersonal skills, motivation, and training, introducing inconsistency in the intervention's delivery.

## Conclusion:

Near-peer and faculty mentorships effectively alleviate burnout among Indian medical students through distinct yet complementary mechanisms. Near-peer mentoring offered empathetic and relatable support, reducing emotional exhaustion, while faculty mentorship provided academic and professional guidance.
